# Controlling the uncontrolled variation in the diet induced obese mouse by microbiomic characterization

**DOI:** 10.1038/s41598-022-17242-8

**Published:** 2022-08-12

**Authors:** Valeriia Bondarenko, Cecillie Reynolds Løkke, Peter Dobrowolski, Caroline Junker Mentzel, Josué L. Castro-Mejía, Camilla Hartmann Friis Hansen, Dorte Bratbo Sørensen, Dennis Sandris Nielsen, Lukasz Krych, Axel Kornerup Hansen

**Affiliations:** 1 Section of Experimental Animal Models, Department of Veterinary and Animal Sciences, Ridebanevej 9, 1871 Frederiksberg C, Denmark; 2 GVG Genetic Monitoring GmbH, Deutscher Platz 5, 04103 Leipzig, Germany; 3 Department of Food Science, Rolighedsvej 26, 1958 Frederiksberg C, Denmark

**Keywords:** Translational research, Microbiome

## Abstract

Group sizes in an animal study are calculated from estimates on variation, effect, power and significance level. Much of the variation in glucose related parameters of the diet-induced obese (DIO) mouse model is due to inter-individual variation in gut microbiota composition. In addition, standard tandem repeats (STRs) in the non-coding DNA shows that inbred mice are not always homogenic. C57BL/6NTac (B6NTac) mice from Taconic and C57BL/6NRj (B6NRj) mice from Janvier Labs were fed a high calorie diet and treated with liraglutide. The fecal microbiota was sequenced before high-calorie feeding (time 1) and after diet-induced obesity instantly before liraglutide treatment (time 2) and mice were divided into clusters on the basis of their microbiota. Although liraglutide in both sub-strains alleviated glucose intolerance and reduced body weight, in a one-way ANOVA a borderline reduction in glycosylated hemoglobin (HbA1c) could only be shown in B6NTac mice. However, if the microbiota clusters from time 1 or time 2 were incorporated in a two-way ANOVA, the HbA1c effect was significant in B6NTac mice in both analyses, while this did not change anything in B6NRj mice. In a one-way ANOVA the estimated group size needed for a significant HbA1c effect in B6NTac mice was 42, but in two-way ANOVAs based upon microbiota clusters of time 1 or time 2 it was reduced to 21 or 12, respectively. The lowering impact on glucose tolerance was also powered by incorporation of microbiota clusters of both times in both sub-strains. B6NRj had up to six, while B6NTac had maximum three alleles in some of their STRs. In B6NRj mice in 28.8% of the STRs the most prevalent allele had a gene frequency less than 90%, while this was only 6.6% in the B6NTac mice. However, incorporation of the STRs with the highest number of alleles or the most even distribution of frequencies in two-way ANOVAs only had little impact on the outcome of data evaluation. It is concluded that the inclusion of microbiota clusters in a two-way ANOVA in the evaluation of the glucose related effects of an intervention in the DIO mouse model might be an efficient tool for increasing power and reducing group sizes in mouse sub-strains, if these have a microbiota, which influences these parameters.

## Introduction

The diet-induced obese (DIO) mouse model, i.e. C57BL/6 (B6) mice fed a high-calorie diet for up to four months of feeding, is one of the most commonly used animal models in research within diabetes and obesity^1^. Compared to a range of other animal models used in biomedical research the model has an acceptable predictive validity, i.e. ability to predict an outcome in humans. For instance, the effect of glucagon-like peptide 1 receptor agonists (GLP-1RA), such as liraglutide, in DIO mice is highly comparable to humans^2^, i.e. it lowers body weight^3^, insulin resistance^4^ and long term blood glucose (HbA1c)^5^. Therefore, these parameters are commonly applied as primary readouts in DIO mice. Glucose tolerance is regarded to be driven by genetics, and, therefore, as the B6 mouse is a highly susceptible inbred mouse strain, it has traditionally been preferred for this model^1,6^. The huge variety of B6 sub-strains can be divided into a main cluster of sub-strains originating from either the Jackson Laboratories (C57BL/6J or B6J) or from the US National Institutes of Health (C57BL/6N or B6N)^7^. Even within these two clusters sub-strains differ genetically^7,8^, and express differences in model phenotype^9,10^. In our own lab, we have experienced that we in some studies can get a significant difference in HbA1c, when using B6NTac from Taconic Biosciences (Ll. Skensved, Denmark), while not when using B6NRj from Janvier Labs (Le Genest-Saint-Isle, France).

DIO mice exhibit a high inter-individual variation^11^. Variation in the response of an animal model reduces power and leads to the need for increased group sizes to show significant differences, as group sizes in animal studies are calculated from estimates on (a) standard deviation (s.d.) and (b) effect size combined with (c) the preferred power (µ) and (d) significance level (*p*)^12^. In some studies the gut microbiota has been shown to account for 30–40% of variation in glucose related parameters, such as HbA1c in the DIO model^13,14^, and the obese phenotype can be transferred between mice by fecal matter transplantation^15^. Therefore, in a mouse study a microbiota impact may be a simple explanation for problems of obtaining enough power and a significant *p*-value for a specific difference. Hypothetically, some B6 mouse colonies may have a microbiota composition, which renders them less sensitive to eliciting certain glucose related responses, such as HbA1c effects. Furthermore, it is generally considered that inbred mice, such as B6, are isogenic, but characterization by short tandem repeats (STRs) reveals that even within a defined sub-strain mice are not genetically homogenic in relation to non-coding DNA^16^. STRs are defined as short runs of repetitive nucleotides and some of them can be relatively polymorphic in the non-coding regions of the DNA, which in both humans and mice represent more than 80% of the genome^17^, and which, although it is not encoding protein transcription, is necessary for transcription of some genes^18^.

Up till today variation in animal models has to a high extent been attempted to be controlled by standardization of factors such as diet and housing conditions^19^. It is, theoretically, possible to standardize an experiment by using the sub-strain most applicable for the purpose, as it for instance has been proposed within modelling for rheumatoid arthritis^20^. However, until recently it was not realized that much inter-individual variation was due to microbiota variation^21^, and how substantially microbiota composition differs between colonies of the same strain^9,10^. As far as the variation is induced by the microbiota, too little is still known to take robust decisions on what would be the most applicable and translational microbiota for diabetes and obesity research. Even mice of the same sub-strain may be delivered by the same vendor but from different breeding rooms with different microbiota^22^. Therefore, a standardization is likely to make studies far more complicated and to decrease reproducibility, and the standardization approach has been questioned, as standardization to one environment may make experiments irreproducible in another environment^23^. It has, therefore, been proposed as an alternative that the variation is systematized to the groups in the experimental design^24^. In a so-called multifactorial heterogenized study the variation is systematically dispersed to each study group and its impact is accounted for in a multifactorial data evaluation, which as a method can be shown to be more reproducible^25^.

Cost of high throughput genomic and microbiomic characterizations are rapidly declining. Microbiomic sequencing becomes increasingly available as a tool. Compared to single nucleotide polymorphisms (SNPs), STRs have a higher mutation rate which benefits the arising of new alleles and makes them highly polymorphic and make them an ideal tool for analysing genetic variability within the same colony of inbred mice^26^. All mice of a study may be characterized and clustered according to their microbiota, and as far as each cluster is represented in each study group, it can be accounted for in multi-factorial data evaluation as for any other factor. Also, the STRs with the highest variability of alleles or a very even distribution of the different alleles may be identified, and the specific allele on a specific STR may be used as a factor in a multifactorial data evaluation. We, therefore, hypothesized that if all DIO mice in an intervention study were characterized according to their individual microbiota and genome, this could be incorporated in data evaluation, and thereby lead to the need for fewer animals or increase the power of a DIO mouse study. We used HbA1c as primary read-out.

## Methods

### Ethics

The experiments were carried out in accordance with the Danish Animal Experiments Act on protection of animals used for scientific purpose (Order 12 of 07/01/2016) and Directive 2010/63/EU of the European Parliament and the Council of 22/09/2010, and the protocol was licensed accordingly by the Animal Experimentation Committee under the Danish Ministry of Food, Fisheries, and Agriculture (License No 2017-15-0201-01262). The study has been reported in accordance with the ARRIVE guidelines^27^.

### Animals

32 C57BL/6NTac (B6NTac; Taconic Biosciences, Lille Skensved, Denmark) and 46 C57BL/6NRj (B6NRj; Janvier Labs, Le Genest-Saint-Isle, France) four week old male mice. Each B6NTac mouse had fecal samples and ears marked at the vendor three days before delivery, while B6NRj had feces sampled at the receiving AAALAC-accredited barrier protected rodent facilities of the Faculty of Health and Medical Sciences, University of Copenhagen (UCPH), Denmark. Here temperatures were 22 °C + 2 °C, 55% ± 10% humidity, and light cycle was 6 am to 6 pm. Mice were housed four per open transparent cage with a wire lid (1290D Eurostandard Type III, Scanbur A/S, Karlslunde, Denmark) with access to bottled tap water ad libitum, aspen bedding (Brogaarden, Lynge, Denmark), nesting material (Brogaarden), a cardboard shelter (Brogaarden), and enrichment including a mini fun tunnel (Brogaarden) and aspen chew block (Brogaarden). Health monitoring of animals was performed without revealing any pathogens according to FELASA guidelines^28^. Cages were changed weekly. To reduce anxiety-related variability in metabolic parameters mice were tunnel handled (from six weeks of age) or cupped without restraint on the hand (from 15 weeks of age) as previously described^29^.

### Experimental design

**Figure 1:**
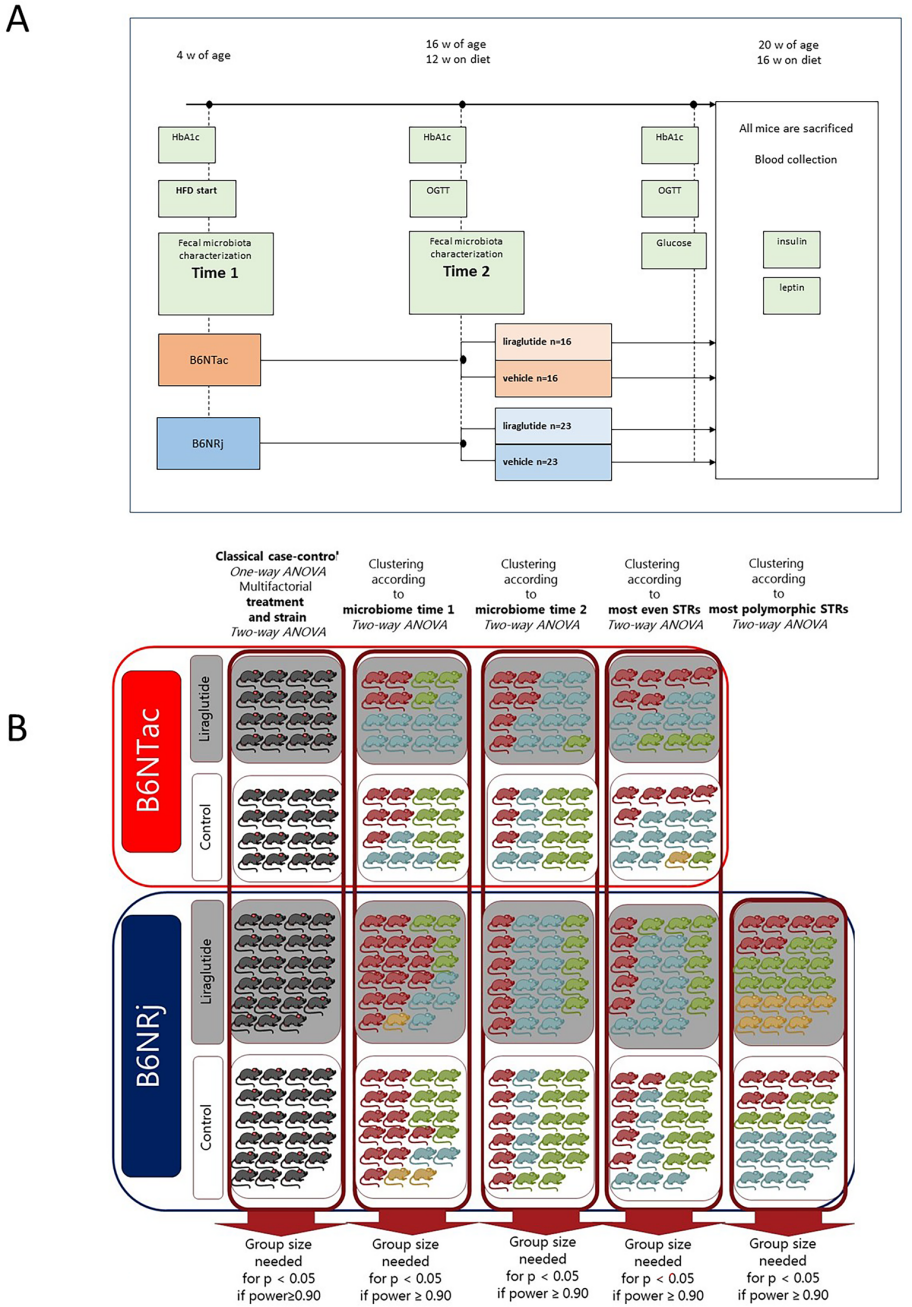
**Overview of the study design.** (**A**) Mice were shipped from the vendor at the age of four weeks. First faecal samples were collected at vendor facility (B6NTac) (mice age 3-4 weeks) or after four days of acclimatization at the experimental facility (B6NRj). First measurement of glycated haemoglobin (HbA1c) was conducted just before mice were started on high-fat diet (HFD). During the next 12 weeks of diet-induced obesity (DIO) body weight (BW) and food intake (FI) were measured weekly. Second faeces samples collection, second HbA1c measurement and first oral glucose tolerance test (OGTT) were conducted during the 12th week of DIO. Intervention with daily subcutaneous injections of liraglutide (0.4 mg/kg) started, when mice were about 16 weeks of age and lasted for 26 days. BW and FI were measured 3 times pr week and all mice were fed HFD during this period. At the end of intervention second OGTT, third measurement of HbA1c and third faecal collection were conducted. On the 27th day of intervention mice were euthanized. Fasting blood glucose was measured before the second OGTT and before euthanasia and retro-orbital blood (for insulin and leptin estimation), caecum content. (**B**) The effect of liraglutide was evaluated in C57BL/6N mice from both Taconic (Tac) and Janvier (Rj) in a classical case-control study. All mice had their fecal microbiota and genome characterized by 16S sequencing and standard tandem repeats, respectively. Statistics were made by simply comparing case (Liraglutide) with control in a one-tailed one-way ANOVA or by incorporation of clusters of microbiome at time points 1 and 2 as a factor in a two-way ANOVA. Based upon observed means and standard deviations the group size needed to obtain p < 0.05 at a power of 0.90 was evaluated. The colours of the mice illustrate the clusters they could be divided into.

Samples sizes were determined by power analysis^12^ on estimated intervention effect and variation in the primary read-out, i.e. HbA1c, based upon in-house experience primarily from B6NTac mice setting *p* < 0.05 and power = 90%. The group sizes for BL6NRj mice were increased with approximately 50%. All mice were fed a synthetic high fat diet (HFD) (Research Diets, Brunswick, USA. Protein: 20% kcal, Fat: 60% kcal, Carbohydrate: 20% kcal) from the age of four weeks to termination (Fig. 1). Food intake per cage and individual body weights were monitored Monday, Wednesday, and Friday. At the age of 16 weeks mice were divided into liraglutide or vehicle treated groups (Taconic mice n = 16/group; Janvier mice n = 23/group) (Fig. 1). For four weeks (age 16–20 weeks) liraglutide treated mice were subcutaneously treated with approximately 0.4 mg/kg liraglutide (as 1:49 dilution of Viktoza®, 6 mg/ml, Novo Nordisk, Denmark) once per day at 15–17 p.m. Mice were treated in a dosing volume of 0.1, 0.125 or 0.15 ml depending on their body weight: 20–30 g, 31–40 g or 41–50 g respectively. Vehicle treated mice (n = 16) received the corresponding amount of sterile filtered (SFCA Nalgene Syringe Filter, Thermo Scientific) dilution buffer (50 mM Na_2_HPO_4_•2H_2_O, 70 mM NaCl and 0.05% polysorbate 80, pH adjusted to 7.5). The first two days of intervention experimental animals received a half dose of liraglutide, i.e. 0.2 mg/kg/day (1:99 dilution), in order to reduce anorexia and adapt animals to the treatment.

### Oral glucose tolerance test (OGTT) and glycated hemoglobin (HbA1c)

Glycated hemoglobin (HbA1c) in mmol/mol was measured at 4 (baseline), 16 (pre-intervention) and 20 (post-intervention) weeks of age in a DCA Vantage Analyzer (Siemens Healthcare Diagnostics, Ballerup, Denmark) by transferring of 1 µL blood sample from the tail vein into the collection cassette supplied. Oral glucose tolerance test (OGTT) was performed before and after intervention with liraglutide (16 and 20 weeks of age). Mice were fasted for six hours (from 7 a.m.) prior to OGTT. Fasting glucose level (t = 0) was measured using a Freestyle Mini Glucometer (Hermedico, Copenhagen, Denmark) by puncture of the tail vein with a 21G needle. It was followed by oral gavage administration of 2 mg/g BW glucose (500 mg/ml glucose solution, Amgro I/S, Copenhagen, Denmark) and subsequent samples of blood glucose level at the times 15, 30, 60, 90 and 120 min.

### Euthanasia

At termination, animals were fasted for six hours. Afterwards, mice were anesthetized with 0.1 ml/10 g BW with 25% Midazolam® (5 mg/ml midazolam, B.Braun, Melsungen, Germany) and 25% Hypnorm® (0.315 mg/ml of fentanyl citrate and 10 mg/ml of fluanisone, Skanderborg Pharmacy, Denmark) diluted in saline. Retro-orbital blood was collected into an autoclaved 1.5 ml Safe-Lock Eppendorf® microcentrifuge tube (Buch & Holm A/S, Herlev, Denmark) and stored on ice for several hours, and later stored at 4 °C for 1 day to let the blood coagulate. The samples were then centrifuged for 10 min at 10,000 rpm at 4 °C. Supernatants were subsequently stored at − 80 °C. Animals were euthanized by cervical dislocation. Caecum contents were collected immediately after euthanizing into sterile Eppendorf® tubes and stored on dry ice during collection, with subsequent storage at − 80 °C. Before post-mortem sample collection mice were randomized, so all parameters were blinded to the reader.

### Insulin and leptin measurements

Fasting serum insulin and leptin levels were measured using the Mouse Metabolic Kit (Meso Scale Diagnostics, Rockville, United States) following the manufacturer’s instructions. Samples from both strains were randomized and measured on one plate. Results were read using MESO QuickPlex SQ 120 (Mesoscale). Leptin measurements with values > 100,000 pg/ml were reported as 100,000 pg/ml due to limits in detection sensitivity of the test.

### Gut microbiota sequencing

Feces for gut microbiota characterization were sampled either at the breeder prior to shipment (B6NTac) or at the experimental facility just before start of feeding period (B6NRj). Collection of feces at time 2 occurred after 12 weeks of high-calorie feeding before intervention with liraglutide. Faecal samples were stored at − 80 °C until further processing. DNA was purified using Bead-Beat Micro AX Gravity kit (A&A Biotechnology, Gdynia, Poland, Cat# 106-100) according to manufacturer’s protocol. Purified DNA was stored at − 80 °C until analysis. DNA concentration and purity were verified on Nano-Drop ND-1000 Spectrophotometer (Thermo Fisher Scientific). Based on Nano-Drop measurements the samples were normalised to 10 ng/µL with nuclease-free water using Biomek 4000 Workstation (Beckman Coulter Life Sciences, United States). The V3 variable region of the bacterial 16S rRNA gene was amplified using the primers NXt_388_F (5′-TCGTCGGCAG CGTCAGATGT GTATAAGAGA CAGACWCCTA CGGGWGGCAGCAG-3′) and NXt_518_R (5′-GTCTCGTGGG CTCG-GAGATG TGTATAAGAG ACAGATTACC GCGGCTGCTGG-3′) compatible with the Nextera Index Kit (Illumina, San Diego, United States). PCR was performed in a final reaction volume of 25 µL composed of 5 µL PCRBIO buffer, 0.5 µL primer mix (10 uM), 0,25 µL PCRBIO HiFi polymerase (PCR Biosystems Ltd, London, UK), 5 µL of genomic DNA, and nuclease-free water up to 25 µL. It was run on SureCycler 8800 (Agilent Technologies, Santa Clara, CA, USA), and the applied thermal cycling conditions were as follows: Enzyme activation for 2 min at 95 °C, 33 cycles of denaturation for 15 s at 95 °C, annealing for 15 s at 55 °C, and extension for 20 s at 72 °C. Lastly, a final extension step for 20 s at 72 °C. Quality of PCR amplicons was assessed by gel electrophoresis using 1.5% agarose gel and 0.5% TBE buffer at 120 V for 40 min. A second PCR was run on SureCycler 8800 to label adapter-ligated DNA fragments with indices. PCR reactions contained 5 µL PCRBIO buffer, 0.25 µL PCRBIO HiFi polymerase, 2 µL of each primer (P5 and P7), 2 µL PCR product, and nuclease-free water to a total volume of 25 µL. Applied thermal cycling conditions were as follows: enzyme activation for 1 min at 95 °C, 13 cycles of denaturation for 15 s at 95 °C, annealing for 15 s and 55 °C, and extension for 15 s at 72 °C. Lastly, a final extension step for 5 min at 72 °C. Labelled adapter-ligated DNA fragments were purified by use of Biomek 4000 Workstation (Beckman Coulter Life Sciences, USA) using AMPure CP binding Beads pooled in equimolar concentrations and sequenced with the Illumina NextSeq550 platform.

The raw dataset with pair-ended reads and corresponding quality scores was merged and trimmed using fastq_mergepairs and fastq_filter scripts implemented in the USEARCH pipeline as described previously^30^. Purging the dataset from chimeric reads and construction of zero radius Operational Taxonomic Units (zOTU) was conducted using the UNOISE. The Ribosomal Database Project (RDP) database (11.5) served as a reference database.

### Genetics

The analysis of tail tips for genotyping was performed by GVG Genetic Monitoring GmbH as previously described^16^. A chromosomal panel of 244 highly polymorphic STR markers evenly distributed in the non-coding regions of the genome was used (Data S2). Snooplex FastPrep PCR reagents (GVG Genetic Monitoring, Leipzig, Germany) in combination with the GVG Genetic Monitoring standard marker panel (GVG Genetic Monitoring) were used for amplification of fluorescence-labeled PCR products. PCR reactions were performed on a Bio-Rad C1000 Touch Thermal Cycler (Bio-Rad Laboratories, Hercules, USA). The standard cycling parameters were 94 °C for 2 min followed by 38 cycles of 94 °C for 30 s, 58 °C for 1 min, 68 °C for 2 min and a final elongation step of 68 °C for 10 min. Amplification products were electrophoresed on an ABI 3500 Genetic Analyzer (Applied Biosystems, Foster City, USA). Briefly, 1.8 μl of amplicons and 12 μl of a 250:1 mixture of deionized formamide and internal lane size standard (MapMarker Custom, BioVentures Inc., Murfreesboro, USA; sizes: 60, 80, 90, 100, 120, 140, 160, 180, 200, 220, 240, 250, 260, 280, 300, 320, 340, 360, 380, 400, 425, 450, 475, 500, 525 and 550 bp labeled with Dy-632) were combined. PCR products were injected (15 s) and electrophoresed at 15 kV in Performance Optimized Polymer 4 (POP4, Applied Biosystems). Data analysis was performed with GeneMapper v5 (Applied Biosystems).

### Statistics

The result of the OGTT was calculated as area under the curve (AUC). For OGTT, body weight and HbA1c the results were expressed as Δ-values, i.e. the difference between pre- and post-intervention values as a percentage of the pre-intervention value (hereafter called the effect). All data were tested for Gaussian distribution by Anderson–Darling test and equal variances by Levene’s test (Minitab 19.0, Minitab Inc., Coventry, UK). For further analysis as factors in two-way ANOVAs all mice were clustered after their microbiome at time 1 and 2 in a group-independent cluster analysis using weighted UniFrac dissimilarities on zOTU tables rarefied to 10,000 reads. The differences between the categories presented with UniFrac based distance matrices were tested with Permutational analysis of variance (PERMANOVA) via vegan: Community Ecology Package in R environment^33^. Hierarchical clustering was based on the Ward’s method and the optimal number of clusters were chosen based on ensuring the maximum possible number of clusters, when each cluster was represented in both treatment and control groups. One-hundred permutations on a generalized-linear model (GML—“lmer4” package R (R Foundation, Vienna, Austria)) were performed and final cluster denotation was as random effect for each permutation. For further analysis as factors in two-way ANOVAs from each sub-strain we selected those STRs, in which there were the most even distribution of alleles within the locus, and those which had the highest number of different alleles within the locus.

All data were compared by both a one-tailed, one-way ANOVA (Minitab: ANOVA/One-Way) as well as two-way ANOVAs with the factors treatment and either cluster or allele (Minitab, ANOVA/General Linear Model/Fit general linear model). For one-way ANOVA the observed differences and standard deviations (s.d.) were used in a power analysis for minimum sample size and power estimation (Minitab, Power and sample size/One-Way ANOVA), while the mean weighted differences and s.d. based upon the differences between treatments and s.d. within each cluster (Table S2) were used for estimation of the minimum sample size and power estimation (Minitab, Power and sample size/General full factorial design) (Fig. 1). Data not following a Gaussian distribution and/or having equal variances were ranked before test.

## Results

### The two sub-strains differed essentially in microbiota composition, and in the B6NRj mice the lack of reads limited the number of mice, which could be clustered

The two sub-strains both before and after treatment differed substantially in their gut microbiota (Figs. S1, S2). Based upon the microbiomic characterization of feces B6NTac could be separated into three clusters at time 1 and 2, while B6NRj could be separated into four clusters at time 1 and 2. One B6NTac mouse at time 2, 8 B6NRj mice at time 1 and 11 B6NRj mice at time 2 did not yield enough reads to be clustered (Data S1).

### Some STRs of B6NRj mice were highly polymorphic

**Figure 2:**
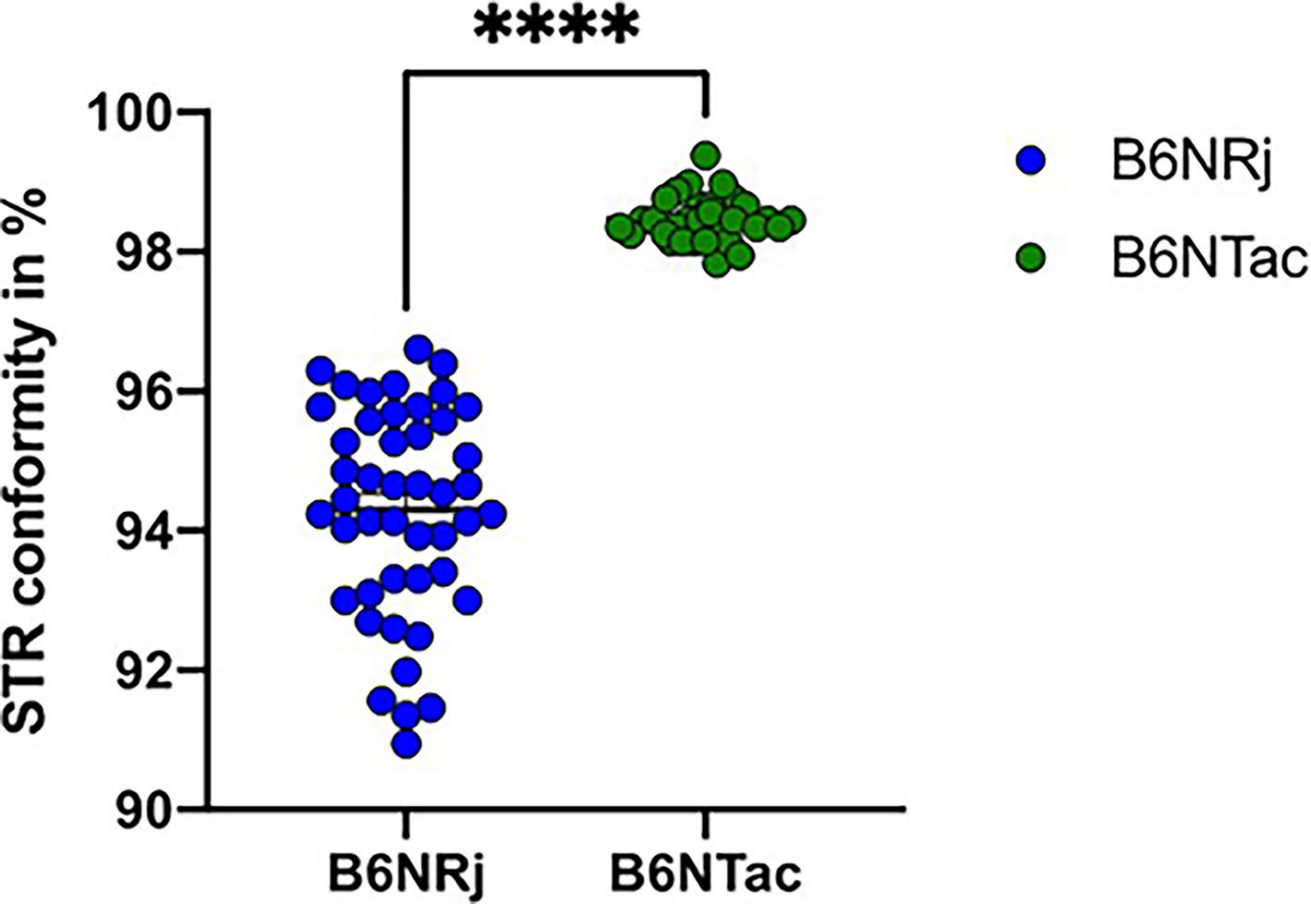
STR conformity in % in C57BL/6NTac and C57BL/6NRj mice. The degree of conformity of the genotype of an individual with the consensus STR-genotype of its strain is calculated by comparing STR alleles for all STR markers. The value of 1 is allocated if both alleles are identical with the consensus allele, a value of 0 if both allels differ and a value of 0.75 if there is a heterozygous match. The resulting sum of all values is then divided by the number of markers and a percentage figure describing the match between the individual and the consensus STR-genotype is obtained. All data are shown as mean ± SEM. **** p<0.0001 ( unpaired t-test with Welch´s correction).

The genetic conformity of the B6NTac mice was significantly higher than the B6NRj mice (*p* < 0.0001, Fig. 2. STRs of B6NTac mice were not polymorphic, as none of them expressed more than three different alleles, while in the B6NRj mice the STRs D9S312, D12S206 and D16S147 expressed five-six different alleles (Table 1). The STRs D1S113, D9S218 and D5S519 showed the most even distribution of the alleles in the B6NTac mice, while the STRs D11S112 and D15S215 showed the most even distribution of alleles in B6NRj mice (Table 1).

**Table 1:** Expression of the most prevalent allele and no of alleles expressed in standard tandem repeats (STRs) of B6NRj and B6NTac mice. STRs written in bold were selected as factors for incorporation in a two-way ANOVA for evaluation of the effect of liraglutide in a model of diet-induced obesity.

	B6NRj STRs N (STR) = 243	B6NTac STRs N (STR) = 243
**Expression of the most prevalent allele in the STRs**		
100%	72 (29.6%)	138 (56.8%)
≥ 90% < 100%	101 (41.6%)	89 (36.6%)
≥ 80% < 90%	36 (14.8%)	10 (4.1%)
≥ 70% < 80%	14 (5.8%)	3 (1.2%)
≥ 60% < 70%	8 (3.29%)	**2 (0.8%)**^**d**^
≥ 50% < 60%	10 (4.1%)	**1 (0.4%)**^**e**^
≥ 40% < 50%	2 (0.8%)^a^	0 (0.0%)
**No. of alleles expressed in the STRs**		
6	**2 (0.8%)**^**b**^	0 (0.0%)
5	**1 (0.4%)**^**c**^	0 (0.0%)
4	7 (2.9%)	0 (0.0%)
3	48 (19.8%)	31 (12.8%)
2	113 (46.5%)	74 (30.5%)
1	72 (29.6%)	138 (56.8%)

^a^D11S112, D15S215; ^b^D12S206, D9S312; ^c^D16S147; ^d^D1S113, D9S218; ^e^DXS312.

Significant values are in bold.

### HbA1c only had a significant effect, if microbiomic clusters were incorporated in data evaluation, and only in B6NTac

HbA1c was only borderline significantly lowered in B6NTac mice (*p* = 0.051, Table 2), while the effect was minimal and non-significant in B6NRj mice (*p* = 0.190, Table 2). When including the microbiomic clusters in a two-way ANOVA there was a significant effect of liraglutide at both time 1 (*p* = 0.033) and 2 (*p* = 0.038) (Table 2). The group size estimated for obtaining significance on the HbA1c effect in B6NTac mice was estimated to be 42 in a one-way ANOVA, while in a two-way ANOVA it was reduced 21, if including the microbiomic clusters from time 1 and 12, if including the microbiomic clusters from time 2 (Table 2). In contrast, there was no effect of liraglutide on HbA1c in the B6NRj mice, which inclusion of the microbiomic clusters in two-way ANOVAs did not change (Table 2).

**Table 2:**
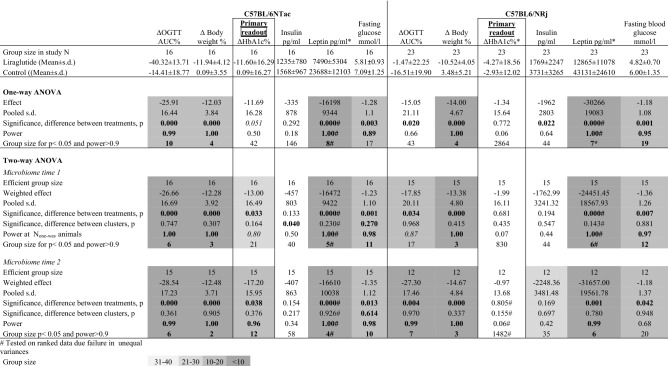
Clinico-chemical parameters from testing the effect of liraglutide in of diet-induced obese C57BL/6NTac mice and C57BL/6NRj and the subsequent evaluation by either one-way ANOVA or two-way ANOVA including treatment and either microbiomic clusters before (time 1) or after diet induction (time 2) a factor. Power is written in bold if > 0.90 and in italics if 0.80 - 0.90.

Significant values are in bold. Italics: borderline significance (p < 0.10).

### Liraglutide had the expected effect on fasting glucose, glucose intolerance, insulin and body weight, which to some extent could be powered by inclusion of the microbiomic clusters in a two-way ANOVA

Liraglutide had the expected effect on glucose intolerance (OGTT) in both B6NTac (*p* = 0.000, Table 2) and B6NRj (*p* = 0.020, Table 2) mice, but 43 B6NRj mice would have been needed in each group to obtain a power of 0.90 (Table 2) and the actual power with 23 mice was only 0.66, while only 10 B6NTac mice would be needed and the actual power with the 16 mice was 0.99 (Table 2). In the B6NRj mice inclusion of the microbiomic clusters of time 2 in a two-way ANOVA decreased the estimated group size needed to show an OGTT effect to 7 and increased the actual power to 0.99 in spite of the fact that the lack of reads at time 2 reduced the efficient group size from 23 to 12 (Table 2). Fasting blood glucose was significantly lowered by liraglutide in both strains (*p* (B6NRj) = 0.001, *p* (B6NTac) = 0.003), and there was some increase in power and decrease in group sizes by including the microbiomic clusters in a two-way ANOVA. This was particularly evident in the B6NTac mice, in which it reduced group sizes and increased power from 17 and 0.89 to 10–11 and 0.98, while in B6NRj mice using time 2 clusters the reduction in efficient group size from 23 to 12 due to the lack of reads seemed to be counterproductive (Table 2). In both strains in a one-way ANOVA the effect on body weight and leptin was that powerful (*p* = 0.000–0.001) that it was difficult to assess whether the two-way ANOVA with the microbiomic clusters had any powering effect (Table 2). Only in B6NRj mice there was a significantly insulin lowering effect of liraglutide (*p* = 0.022, Table 2).

### Including selected STRs in a two-way ANOVA increased power and decreased estimated group size for evaluating insulin in B6NRj mice

In neither of the sub-strains the inclusion of the selected STRs as factors in a two-way ANOVA had a major impact on the power and the estimated group sizes (Tables 3, 4). Most remarkably inclusion of D12S206 resulted in a *p* = 0.006, a power of 0.93 and an estimated group size of 21 needed for evaluation of insulin compared to *p* = 0.022, power = 0.64 and an estimated group size of 44 if based upon a one-way ANOVA (Table 4).

**Table 3:**
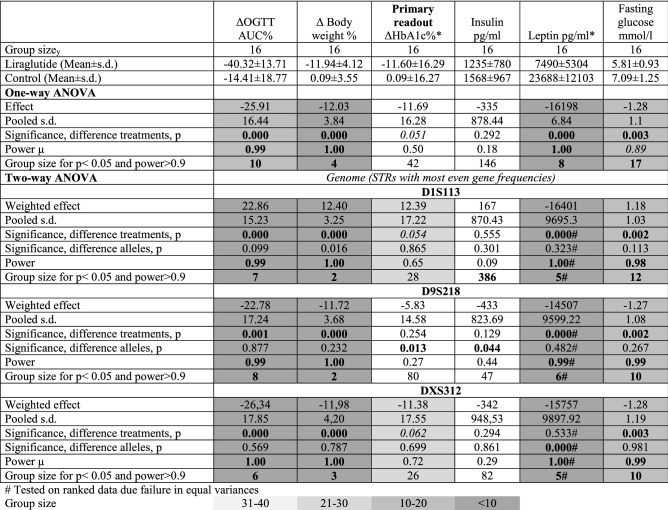
Clinico-chemical parameters from testing the effect of liraglutide in diet-induced obese C57BL/6NTac mice and the subsequent evaluation by either one-way ANOVA or two-way ANOVA including alleles with the most even gene frequencies as a factor. Group sizes are written in bold if they are lower than the actual efficient group size. p < 0.05 are written in bold, p = 0.05 -0.010 are written in italics. Power is written in bold if > 0.90 and in italics if 0.80 - 0.90.

Significant values are in bold. Italics: borderline significance (p < 0.10).

**Table 4:**
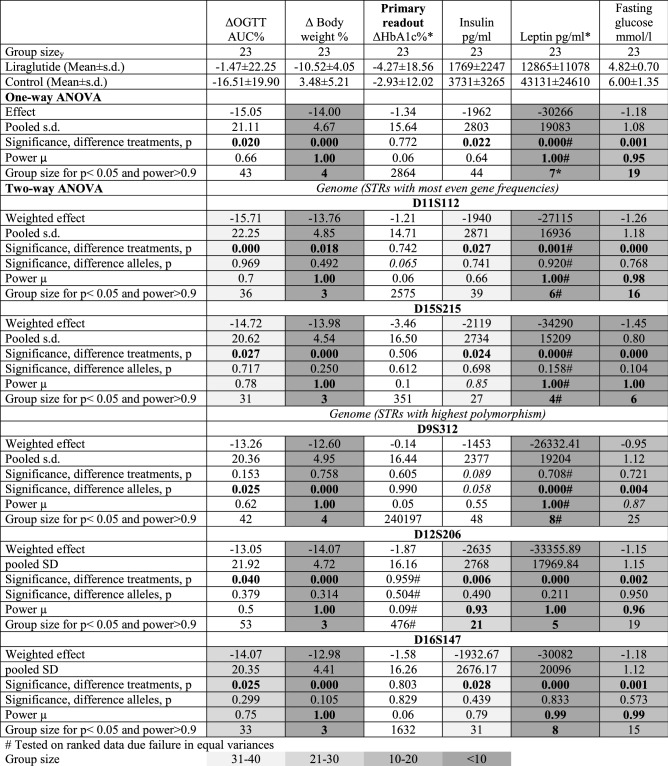
Clinico-chemical parameters from testing the effect of liraglutide in diet-induced obese C57BL/6NRj mice and the subsequent evaluation by either one-way ANOVA or two-way ANOVA including either alleles with the most even gene frequencies or alleles with the highest polymorphism as a factor. Group sizes are written in bold if they are lower than the actual efficient group size. p < 0.05 are written in bold, p = 0.05 -0.010 are written in italics. Power is written in bold if > 0.90 and in italics if 0.80 - 0.90.

Significant values are in bold. Italics: borderline significance (p < 0.10).

## Discussion

Since the late fifties, it has been regarded an obligation whenever using animals for research to reduce the number of animals used in individual studies as one of the three 3 R’s, i.e. replacement, reduction and refinement^35^. This study has shown that inclusion of the microbiota clusters from a sequencing based characterisation as a factor in a two-way ANOVA increases power in some key parameters of the DIO model. By this method, we increased the chances of showing a liraglutide lowering impact on HbA1c in the B6NTac mice. In contrast, the effect in B6NRj mice was either absent or minimal, and considering the microbiota characterisation in a two-way ANOVA did not influence this. However, this strengthens the conclusion that there probably is no effect of liraglutide on HbA1c in B6NRj mice, although this is not predictive for humans. As HbA1c in some mice seems strongly correlated to gut microbiota composition^13^, a reasonable explanation could be that a deviating microbiota makes B6 mice from some colonies, such as the B6NRj mice, rather resistant to HbA1c changes. The two sub-strains differed qualitatively and quantitatively in key bacterial species relevant for obesity and glucose intolerance in both mice and humans^14,36–40^, such as *Bifidobacterium* spp., *A. muciniphila*, Lactobacillaceae, *Bacteroides* spp. and Prevotellaceae.

Strong microbiota impact has also been described for a number of animal models within a broad spectrum of other biomedical disciplines^41,42^, such as brain-related^43,44^, autoimmune^45^, and allergic^46^ diseases, as well as solid organ transplant outcomes^47^, blood pressure^48^, acute pancreatitis^49^, colorectal cancer^50^ and others. Therefore, it seems as if the microbiome composition also influences results of mouse studies not previously thought to be influenced by it. For example, it is a novel insight in cancer research that microbiota may change the response to chemotherapy^51^, in schizophrenia research that one of the most commonly applied rat models can be modified by antibiotics^52^, and in diabetes research that metformin may exert part of its hypoglycemic effects by altering the gut microbiota^53^. Therefore, our approach may be applied to increase power in a range of models. The further optimization of these tools based on multiple parameters, such as distance type, data normalization, and clustering approaches, for a better control with and incorporation of the microbiota variation in the data evaluation, would likely make them more applicable for the presented as well as for other models.

HbA1c is very much correlated to the rate of erythrocyte turnover, and this may be influenced also by factors such as nutritional status and genetics^54^. The microbiota of mice is heavily influenced by feeding^55^, sub-strains of B6 mice differ genetically^8^ and mouse populations have their own unique core microbiota^56^, and, therefore, differences in HbA1c response between B6 sub-strains is likely to be multifactorial.

As liraglutide lowers HbA1c in humans^5^, B6NTac compared to B6NRj seems to have a higher predictive validity. Still, liraglutide induced lower body weight and increased glucose intolerance, which is known from humans^3,4^, could be shown in both B6NTac and B6NRj mice even in a one-way ANOVA. Inclusion of the microbiota clusters in a two-way ANOVA also increased the power of showing an impact on OGTT in the B6NTac mice.

It is critical for the organization of the mice into clusters that there is a sufficient number of reads in the sequencing for each mouse. The reduction in efficient group sizes due to the lack of reads in B6NRj mice made the method less applicable for the parameters of fasting blood glucose and insulin. In the calculation of group sizes and power for the multifactorial evaluation, we used the weighted average effect in the equation. This is very conservative as group sizes and power are determined by the maximum effect. However, the use of a more conservative estimate may lead to a less optimistic but eventually more reachable estimate of group sizes, as one cannot always expect any maximum effect to be universally reproducible. However, the effect of microbiota incorporation may be even more favourable than the estimates we have made.

The B6NRj mice were more genetically heterogenous than the B6NTac mice. However, our two-way ANOVAs did not indicate that this heterogeneity could explain the lowered or lacking effect of liraglutide on OGTT and HbA1c in the B6NRj mice. With only 29.6% of the B6NRj STRs being 100% homozygous this sub-strain may not be regarded in full accordance with what would be expected from an inbred mouse. However, in 86% of the B6NRj STRs the most prevalent allele had a frequency of more than 80%. Therefore, most likely this genetic heterogeneity is more to be regarded as a trace contamination without the same impact on the parameter responses as the microbiota fluctuations.

In conclusion, the inclusion of microbiota clusters in a two-way ANOVA in the evaluation of the glucose related effects of an anti-diabetic and anti-obesity intervention in the DIO mouse model is an efficient tool for increasing power and reducing group sizes in B6 mouse sub-strains, as far as they have a microbiota, which influences these parameters.

## Supplementary Information

Supplementary Information 1.

Supplementary Information 2.

Supplementary Information 3.

Supplementary Information 4.

Supplementary Information 5.

## Data Availability

Clinico-chemical data and scripts are available from the Open Science Framework on https://osf.io/fhgwt/. Microbiomic data are available at https://www.ncbi.nlm.nih.gov/bioproject/752003.
