# Supplementary material for: Controlling the uncontrolled variation in the diet induced obese mouse by microbiomic characterization
Source: Sci Rep. 2022 Aug 12;12:13767. doi: 10.1038/s41598-022-17242-8 (PMC9374709; doi:10.1038/s41598-022-17242-8)
Supplement: Supplementary file 3 — Supplementary Information 3. [file 41598_2022_17242_MOESM3_ESM.pptx]

## Slide 1
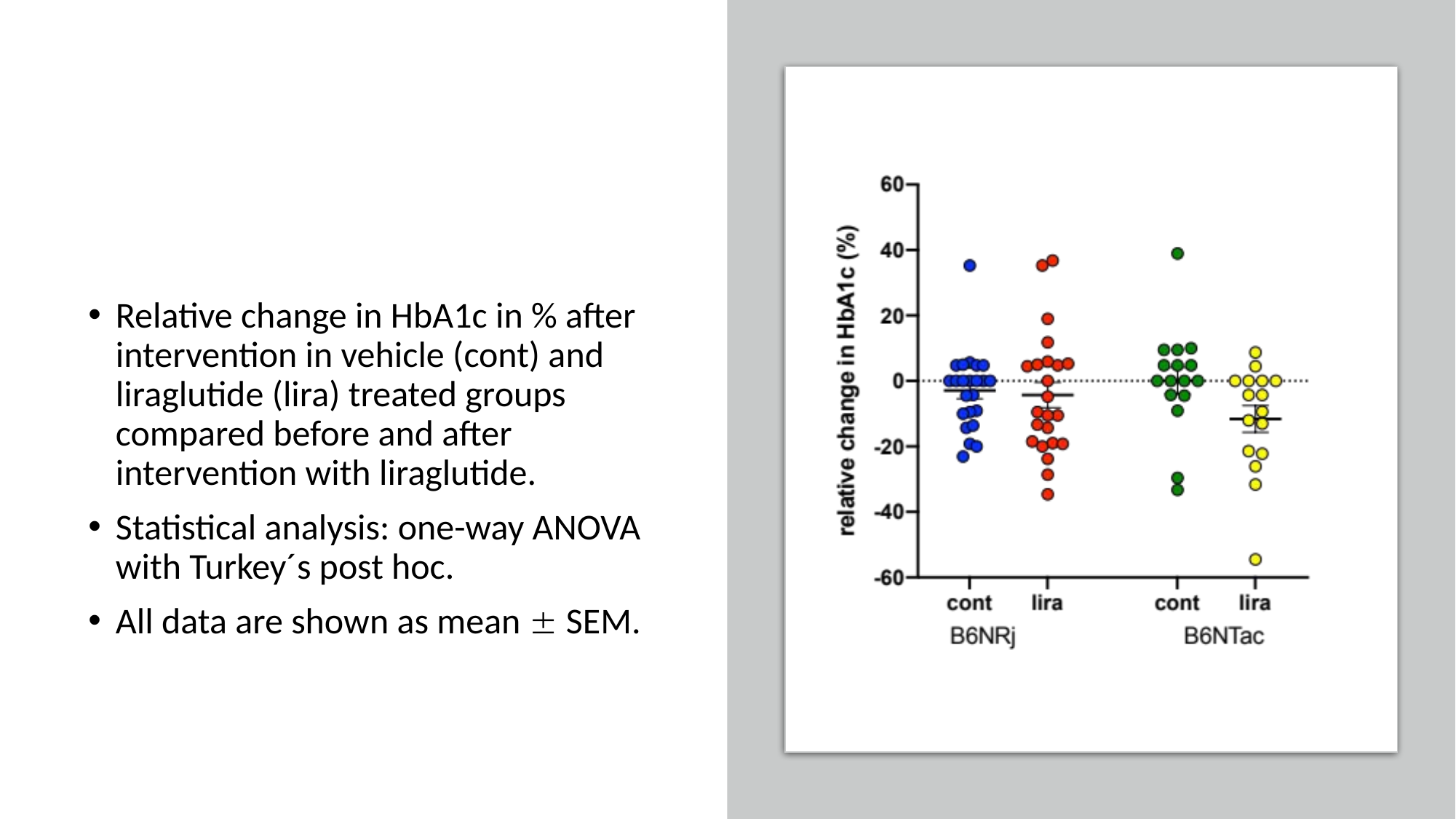

Relative change in HbA1c in % after intervention in vehicle (cont) and liraglutide (lira) treated groups compared before and after intervention with liraglutide.
Statistical analysis: one-way ANOVA with Turkey´s post hoc.
All data are shown as mean ± SEM.

## Slide 2
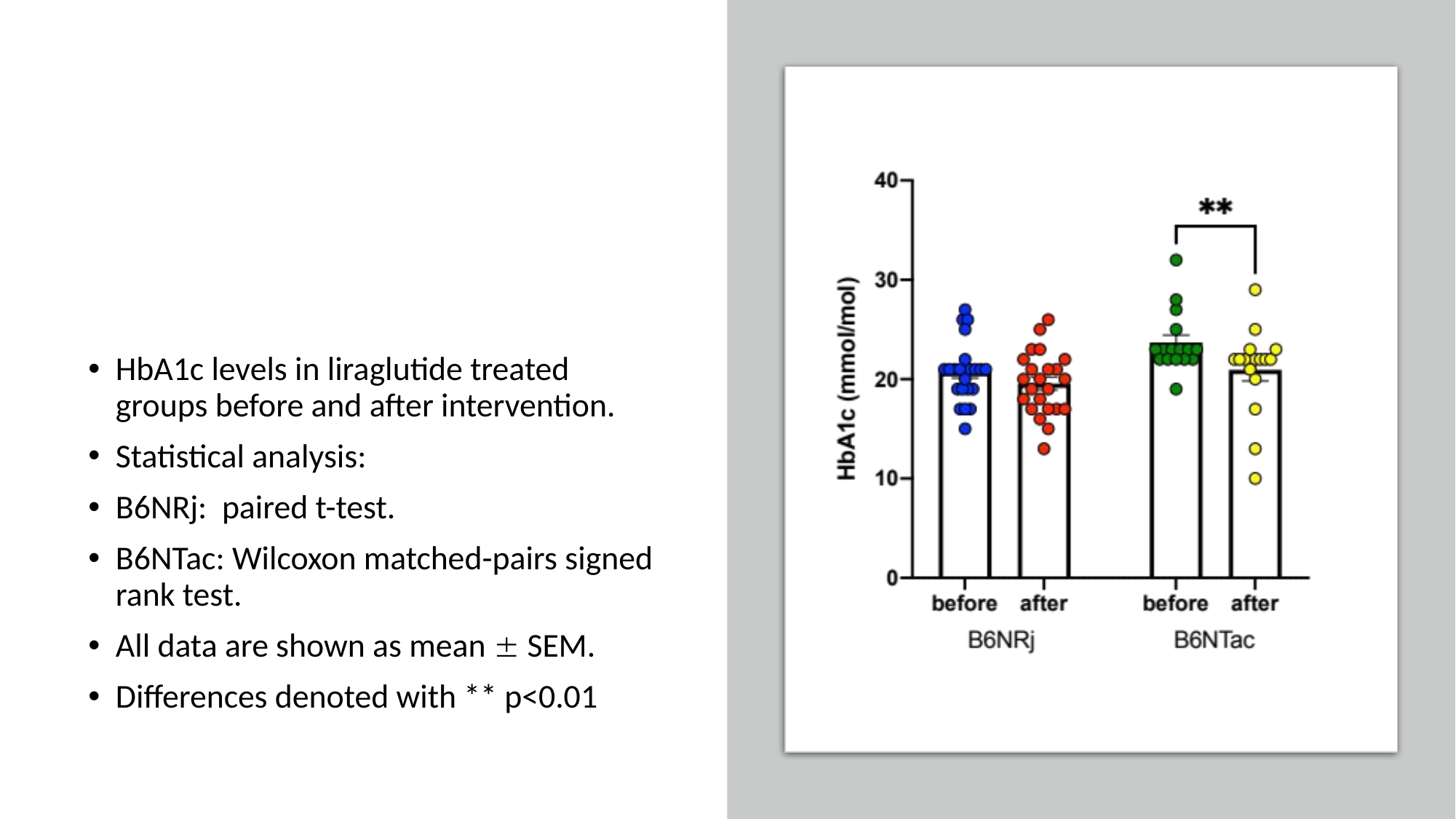

HbA1c levels in liraglutide treated groups before and after intervention.
Statistical analysis:
B6NRj: paired t-test.
B6NTac: Wilcoxon matched-pairs signed rank test.
All data are shown as mean ± SEM.
Differences denoted with ** p<0.01

## Slide 3
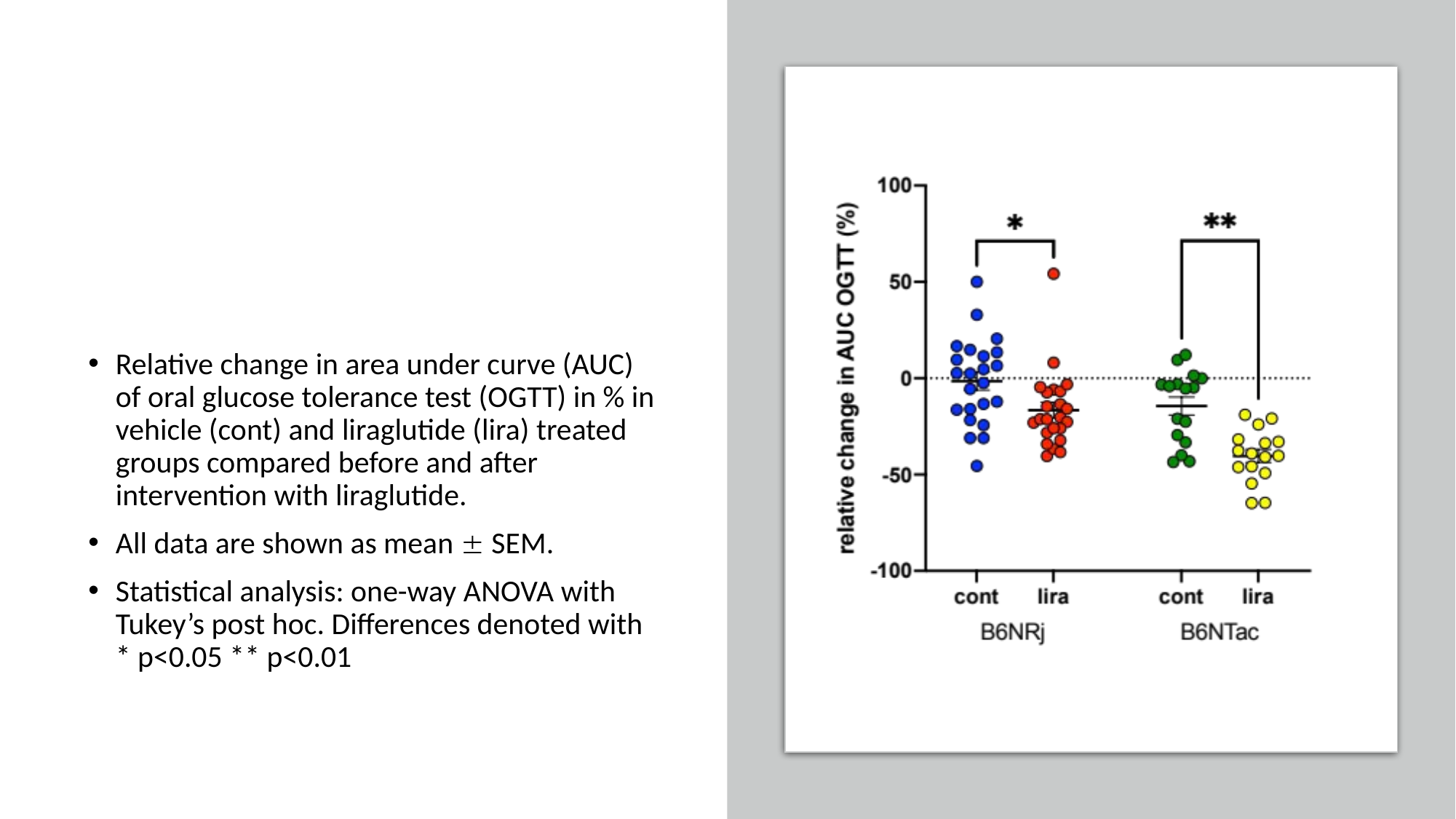

Relative change in area under curve (AUC) of oral glucose tolerance test (OGTT) in % in vehicle (cont) and liraglutide (lira) treated groups compared before and after intervention with liraglutide.
All data are shown as mean ± SEM.
Statistical analysis: one-way ANOVA with Tukey’s post hoc. Differences denoted with * p<0.05 ** p<0.01

## Slide 4
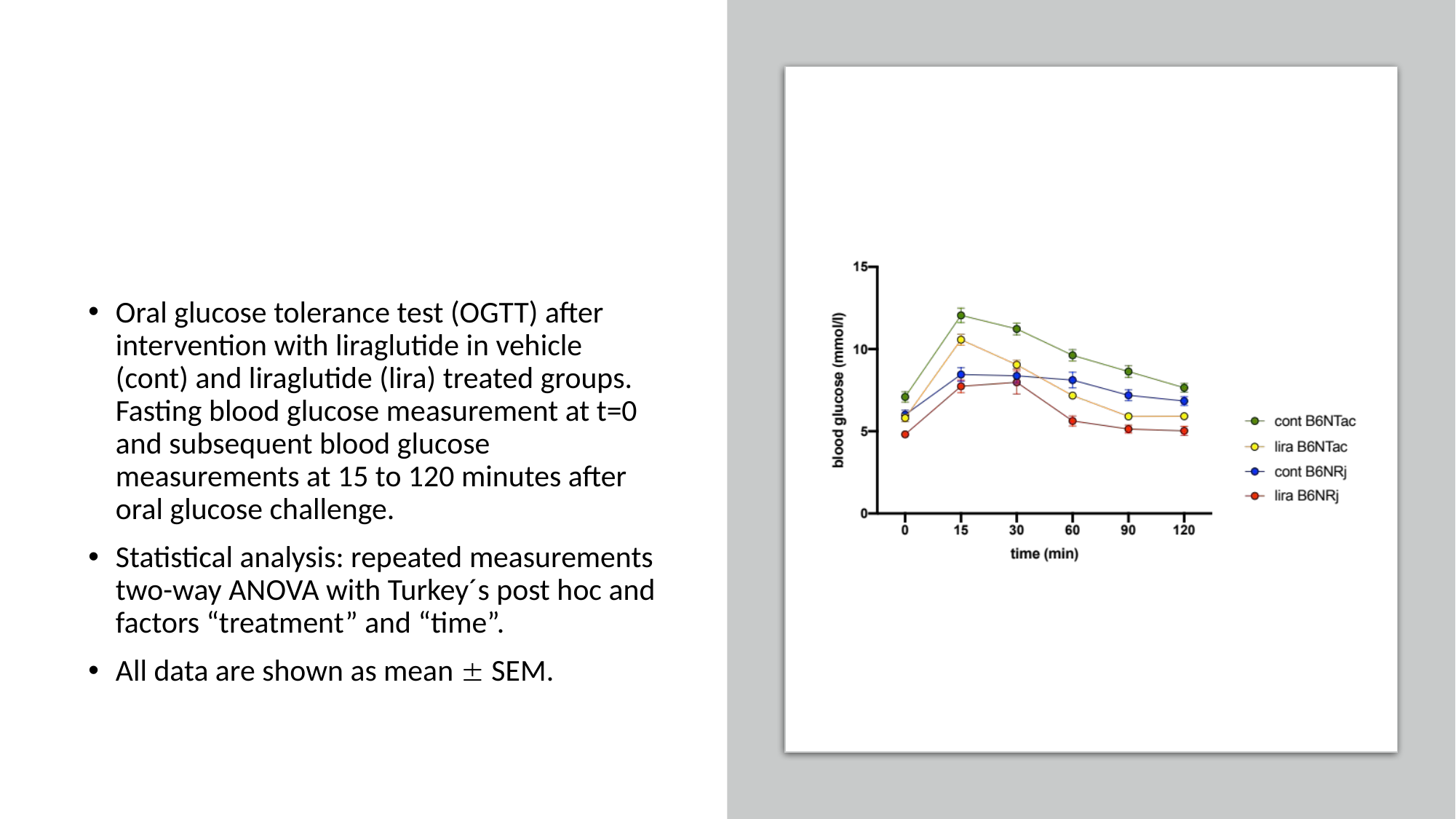

Oral glucose tolerance test (OGTT) after intervention with liraglutide in vehicle (cont) and liraglutide (lira) treated groups. Fasting blood glucose measurement at t=0 and subsequent blood glucose measurements at 15 to 120 minutes after oral glucose challenge.
Statistical analysis: repeated measurements two-way ANOVA with Turkey´s post hoc and factors “treatment” and “time”.
All data are shown as mean ± SEM.

## Slide 5
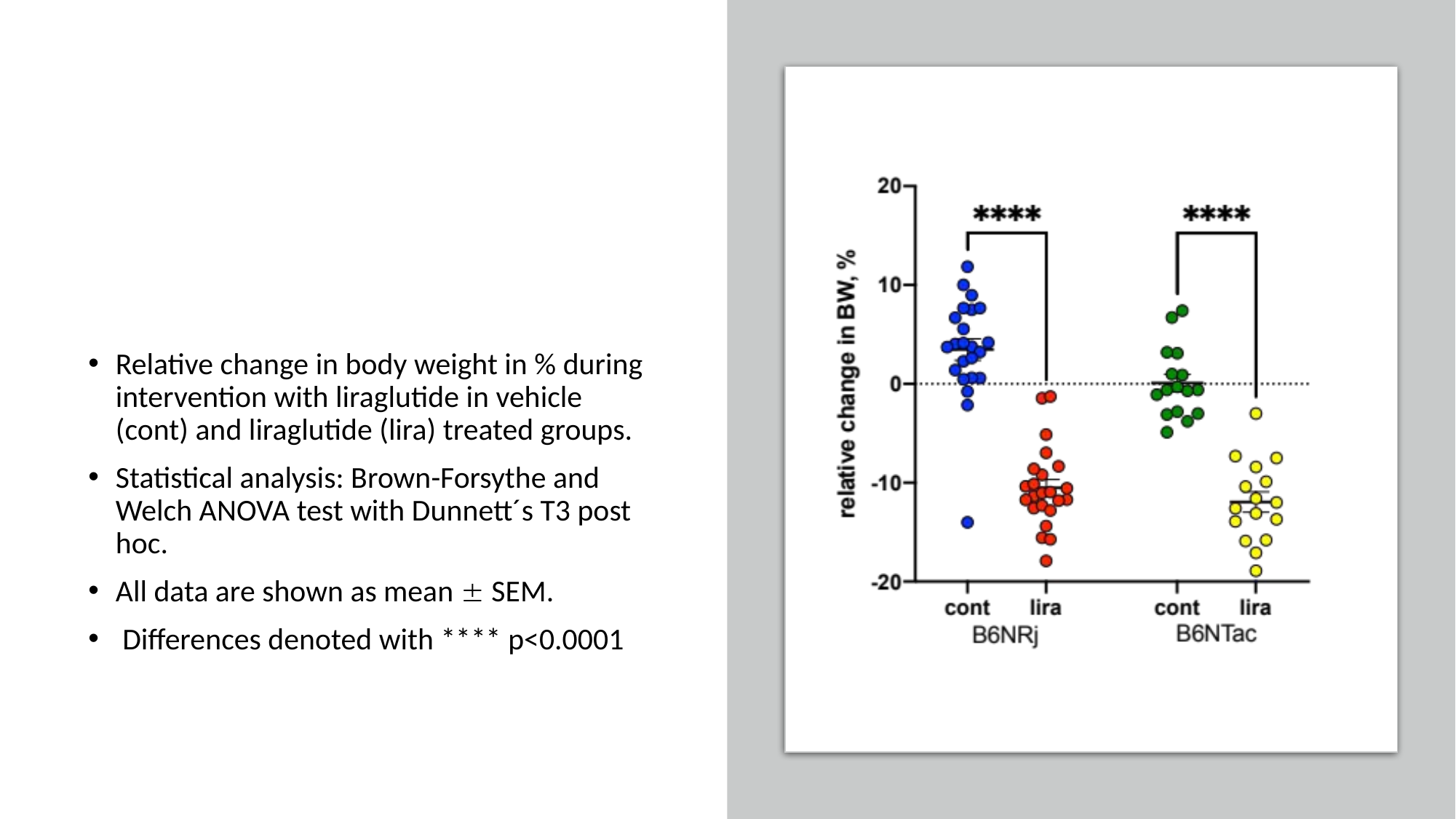

Relative change in body weight in % during intervention with liraglutide in vehicle (cont) and liraglutide (lira) treated groups.
Statistical analysis: Brown-Forsythe and Welch ANOVA test with Dunnett´s T3 post hoc.
All data are shown as mean ± SEM.
 Differences denoted with **** p<0.0001

## Slide 6
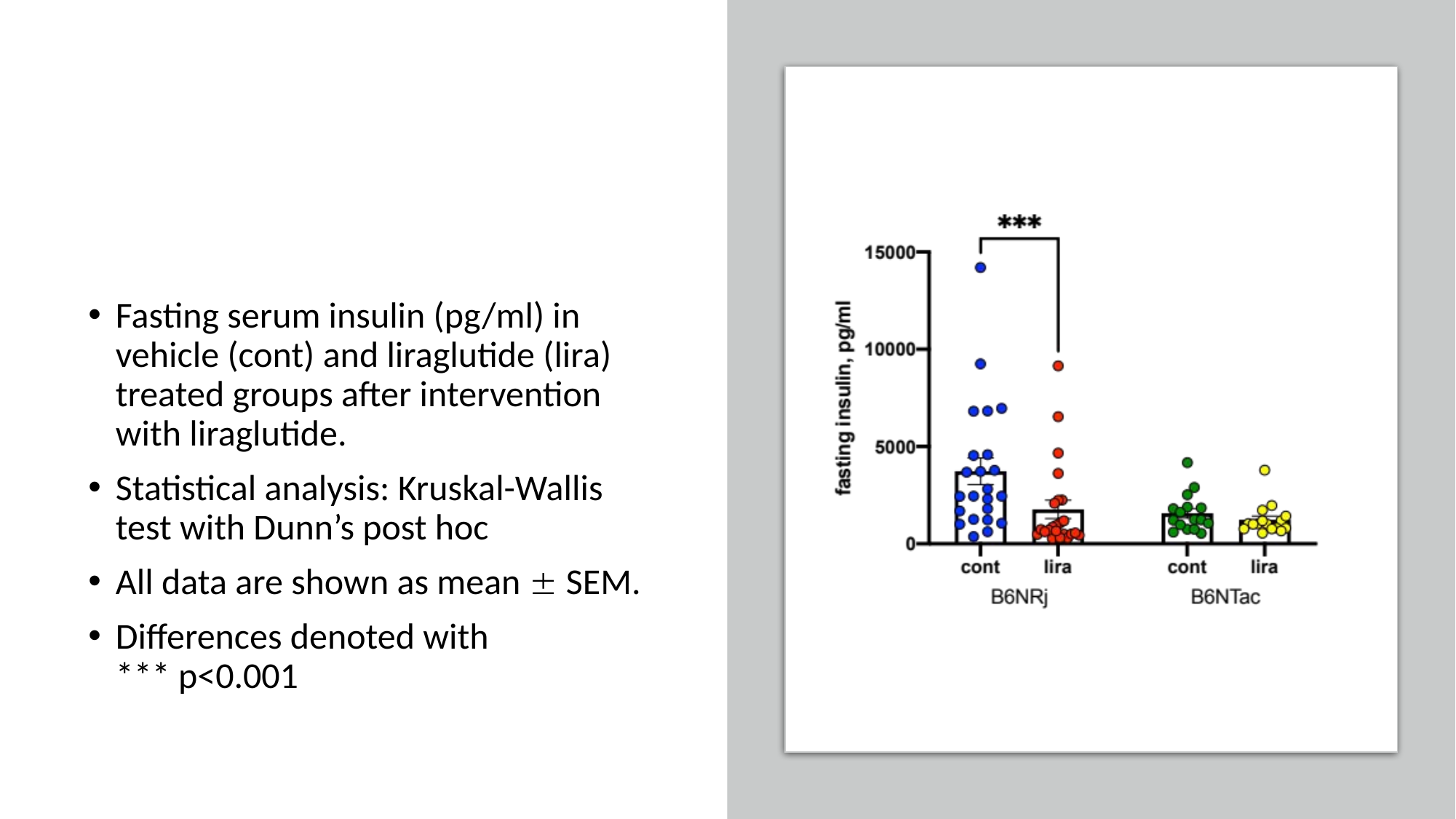

Fasting serum insulin (pg/ml) in vehicle (cont) and liraglutide (lira) treated groups after intervention with liraglutide.
Statistical analysis: Kruskal-Wallis test with Dunn’s post hoc
All data are shown as mean ± SEM.
Differences denoted with *** p<0.001

## Slide 7
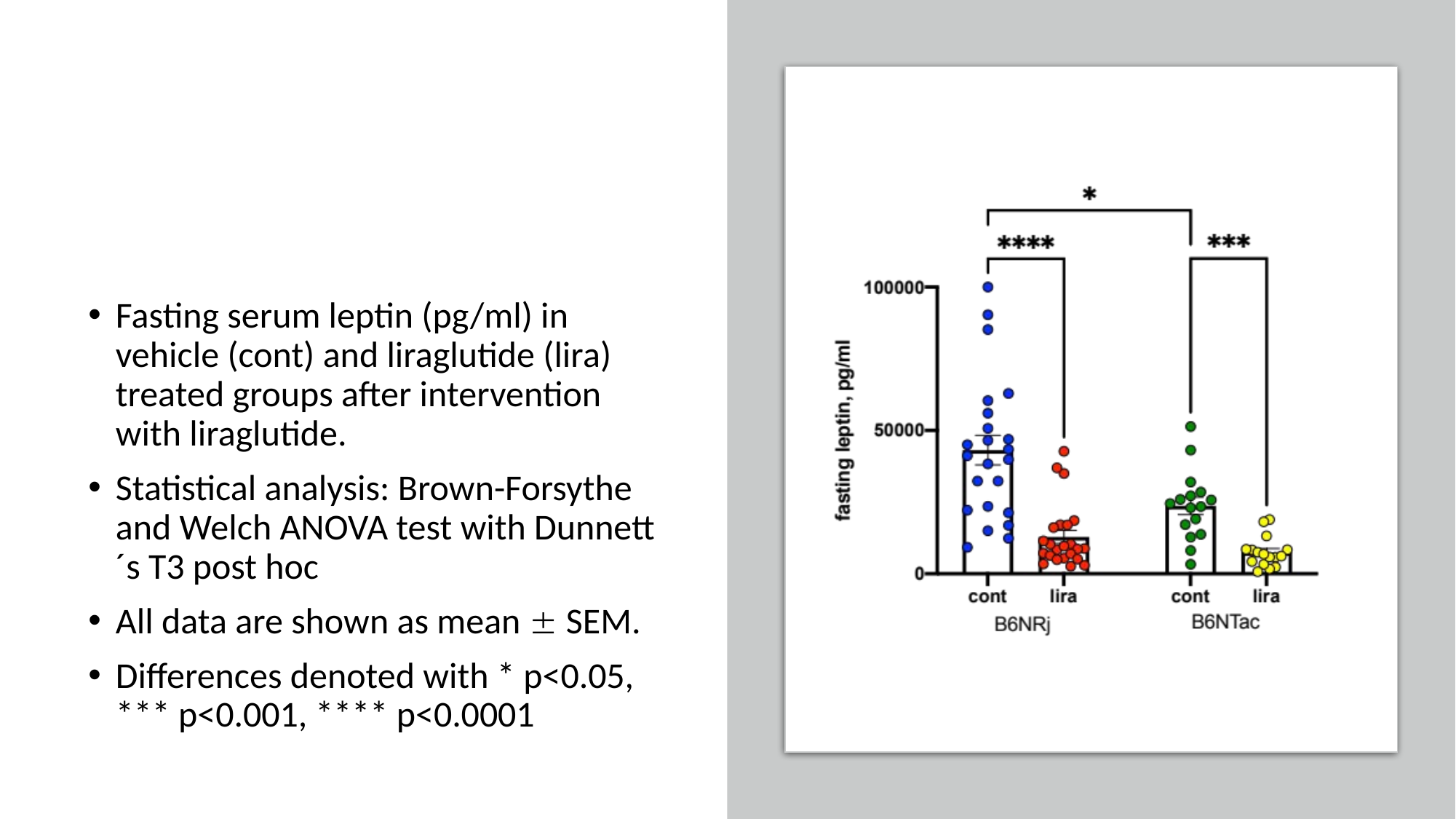

Fasting serum leptin (pg/ml) in vehicle (cont) and liraglutide (lira) treated groups after intervention with liraglutide.
Statistical analysis: Brown-Forsythe and Welch ANOVA test with Dunnett´s T3 post hoc
All data are shown as mean ± SEM.
Differences denoted with * p<0.05, *** p<0.001, **** p<0.0001

## Slide 8
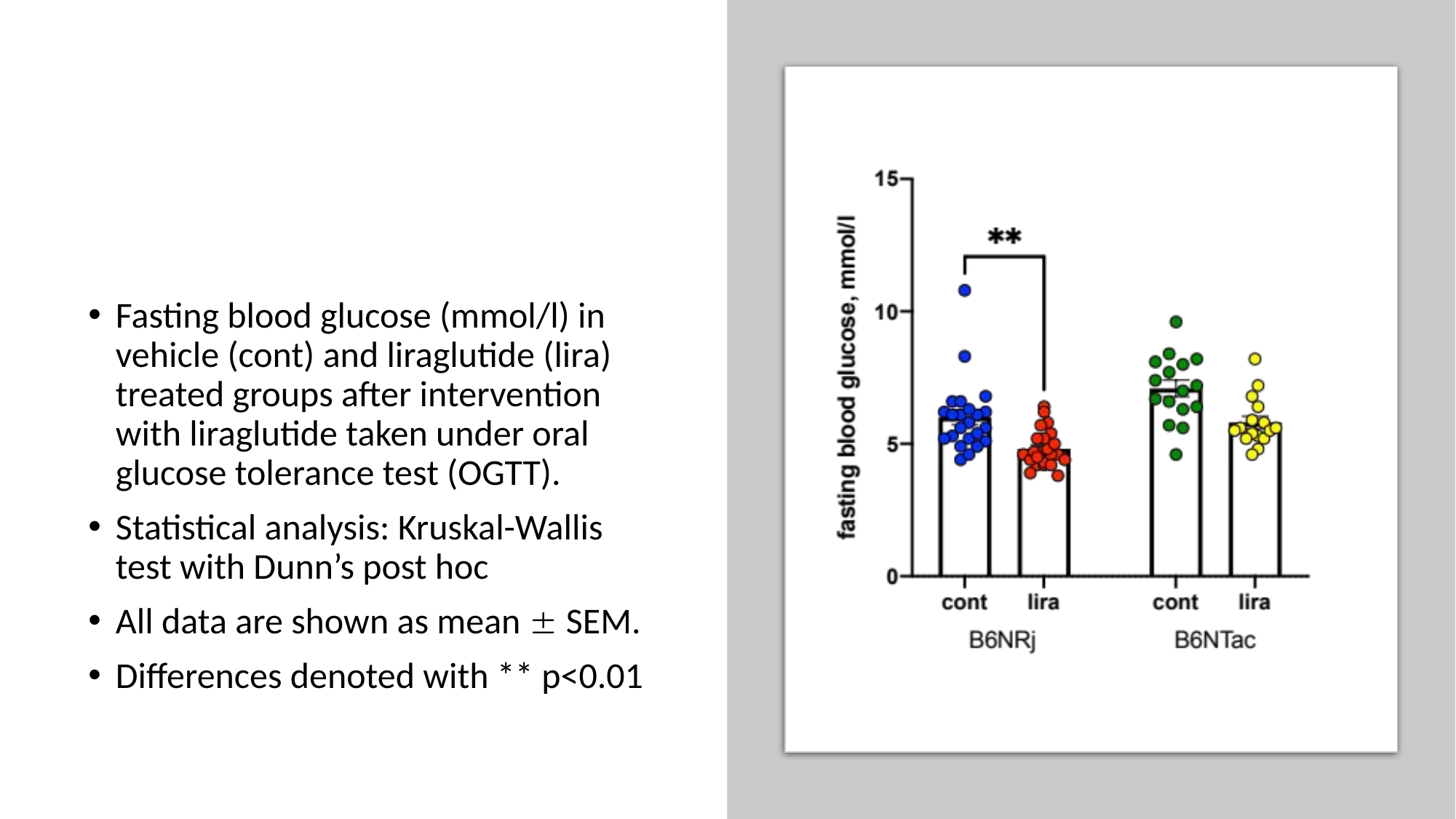

Fasting blood glucose (mmol/l) in vehicle (cont) and liraglutide (lira) treated groups after intervention with liraglutide taken under oral glucose tolerance test (OGTT).
Statistical analysis: Kruskal-Wallis test with Dunn’s post hoc
All data are shown as mean ± SEM.
Differences denoted with ** p<0.01
