# Supplementary figures and images for: Controlling the uncontrolled variation in the diet induced obese mouse by microbiomic characterization
Source: Sci Rep. 2022 Aug 12;12:13767. doi: 10.1038/s41598-022-17242-8 (PMC9374709; doi:10.1038/s41598-022-17242-8)

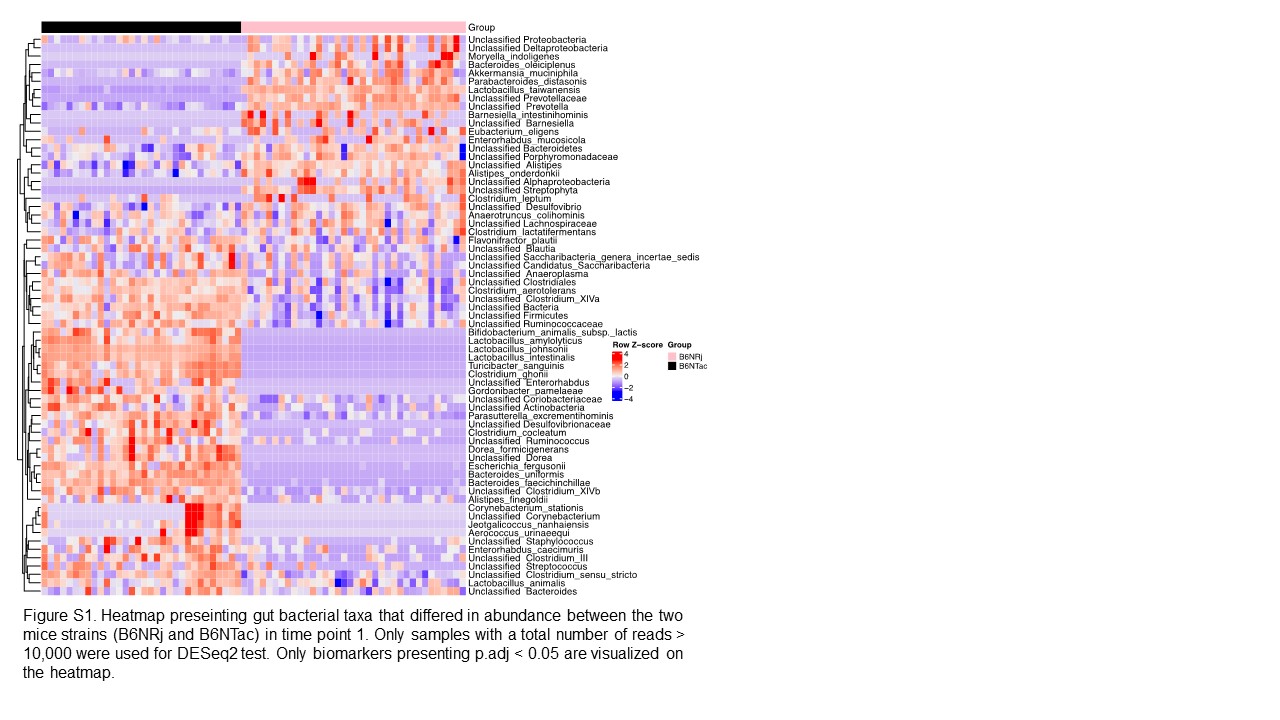

Supplement: Supplementary file 4 — Supplementary Information 4. [file 41598_2022_17242_MOESM4_ESM.jpg]

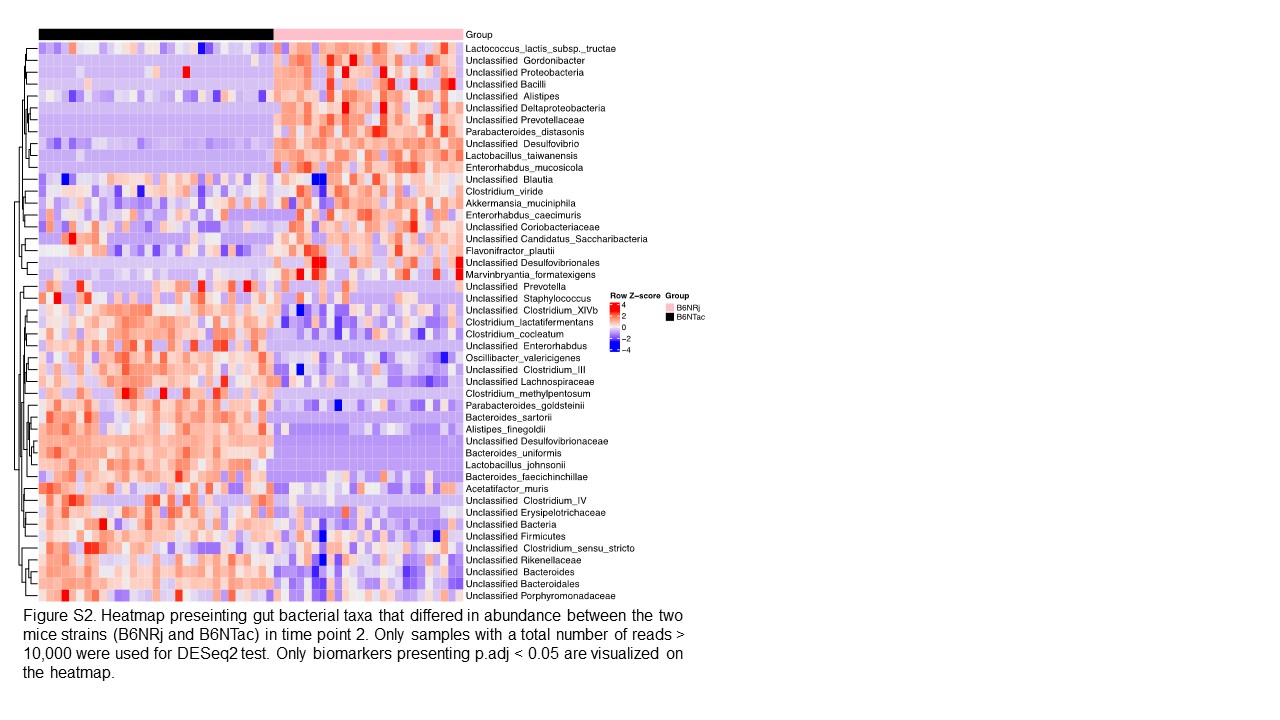

Supplement: Supplementary file 5 — Supplementary Information 5. [file 41598_2022_17242_MOESM5_ESM.jpg]
